# Atypical autism in a boy with double duplication of 22q11.2: implications of increasing dosage

**DOI:** 10.1038/s41525-017-0031-6

**Published:** 2017-09-28

**Authors:** Breanne Dale, Bonnie MacKinnon Modi, Sanne Jilderda, Beth McConnell, Ny Hoang, Pooja Swaroop, Jhoan Falcon, Bhooma Thiruvahindrapuram, Susan Walker, Stephen W. Scherer, D. James Stavropoulos, Irene E. Drmic, Melissa T. Carter

**Affiliations:** 1 Autism Research Unit, The Hospital for Sick Children, Toronto, ON Canada; 2 The Centre for Applied Genomics, Hospital for Sick Children and McLaughlin Centre and Department of Molecular Genetics, University of Toronto, Toronto, ON Canada; 3 Department of Pathology and Laboratory Medicine, Genome Diagnostics, The Hospital for Sick Children, University of Toronto, Toronto, ON Canada; 4 Department of Pediatrics, Division of Clinical and Metabolic Genetics, The Hospital for Sick Children, Toronto, ON Canada; 5 Regional Genetics Program, The Children’s Hospital of Eastern Ontario, Ottawa, ON Canada

## Abstract

Duplication of chromosome 22q11.2 (LCR A-D) has been reported at higher frequencies in clinical samples than the general population, but phenotypes vary widely. Triplication (4 copies) is rare, but studying the associated phenotype may provide insight into dosage-sensitivity of the genes in this chromosomal interval. We describe a proband with a triplication, specifically a “double duplication” (two copies per chromosome) of the 22q11.2 region, while his parents and two siblings each have a single duplication (3 copies). The proband had a heart malformation, dysmorphic features, and learning and socialization deficits, whereas the other family members did not. This family illustrates that while duplication of the 22q11.2 may not be sufficient to cause clinically significant neurodevelopmental or health-related phenotypes, triplication of the same region may result in a phenotype characterized by a mild neurodevelopmental disorder, facial dysmorphism, and possibly cardiac anomalies.

## Introduction

The 22q11.2 chromosomal region is susceptible to rearrangements due to the presence of several low-copy repeat sequences.^1^ These sequences facilitate non-allelic homologous recombination (NAHR) between chromosomes leading to deletions and duplications, typically 1.5 to 3 megabases (Mb) in length.^1^ The 22q11.2 deletion syndrome has long been recognized as a heterogeneous condition characterized by congenital heart defect, cleft palate or velopharyngeal insufficiency, developmental delay, and a heightened predisposition to psychiatric disorders.^2^ The first individual with a duplication of the 22q11.2 region (3 copies) detected by fluorescence in situ hybridization (FISH) was described in 1999.^3^ Since then, over 50 additional cases have been reported, with highly variable features.^4^ Early reports were identified by FISH analysis for suspicion of 22q11.2 deletion syndrome (22q11.2 DS), and therefore were biased towards those with similar features.^5^ Clinical features reported in individuals ascertained by chromosomal microarray (CMA), in contrast, are often mild and highly variable, including cognitive and behavioral issues, congenital malformations, facial dysmorphism, and other health problems.^4^ The duplication is inherited from a parent in at least 70% of reported cases.^4^

Here we describe a family of five, ascertained via the proband with atypical autism who carries a triplication (4 copies) of the 22q11.2 region, specifically two copies on each chromosome; herein referred to as “double duplication” (2:2). Both parents and the unaffected siblings carry the 22q11.2 duplication (2:1). To our knowledge, this is the first reported case of 22q11.2 double duplication. We describe in detail the clinical features of the proband, who clinically resembles a previously reported child with a triplication (3:1) at 22q11.2.

## Results

### Molecular characterization of family

CMA in the proband showed four copies of the 22q11.2 region from nucleotide position 18,890,162 to 21,440,515 (hg19). Genotype analysis confirmed the parental self-reporting of non-relatedness to each other (data not shown). The father has a copy number gain at 22q11.21 of 2.73 Mb from nucleotide position 18,713,432 to 21,440,515 (hg19) and the mother has a copy number gain at 22q11.21 of 2.55 Mb from 18,890,162 to 21,440,515 (hg19). Both siblings of proband had similar gains (Supplementary Figure 1). Copy number variant (CNV) analysis examining sequence read-depth of whole genome sequencing (WGS) data confirmed the duplications (data not shown). FISH testing of both parents confirmed a 2:1 configuration of the copy number gains at 22q11.2 and a 2:2 configuration in the proband (Supplementary Figure 1). Using the WGS data, the allele ratio of informative SNP genotypes within the 22q11 duplication (where both parents are homozygous, but had different genotypes with the proband being heterozygous), confirmed this interpretation (Supplemental information Fig. 2).

Whole genome sequencing of the proband's DNA identified putative loss-of-function variants or compound heterozygous variants in the autism spectrum disorder (ASD) candidate genes *MSR1* and *SDC2*, as well as *MYO1A* and *CLTCL1*, respectively. Moreover, a de novo frameshift mutation in *SMAD6* (p.Ala424Profs*115) was observed (all mutations are described in Supplemental information Table 1). Using methods described previously^6, 7^, no additional rare coding CNVs were detected.

### Clinical characterization of the family

#### II-1: Proband (double duplication 22q11.2)

**Fig. 1:**
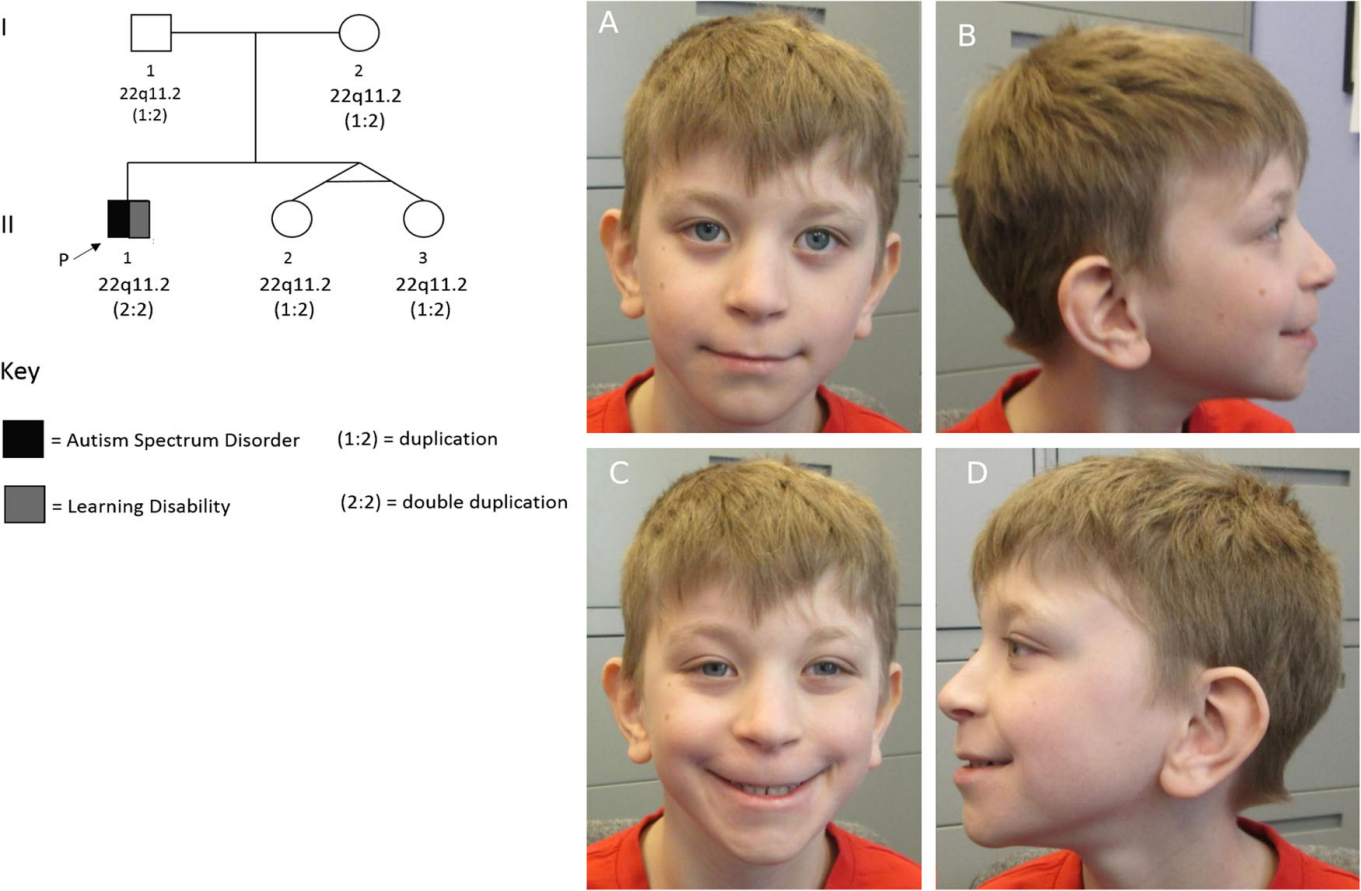
Pedigree of family and facial photographs of proband (II-1). The parents provided written informed consent for publication of photographs. Note distinctive facial features including hooded eyelids **a**, long and smooth philtrum with a thin upper lip **a** and **c**, low-set and posteriorly rotated ears **b** and **d** with preauricular pits and forward facing earlobes

The proband (II-1 in Fig. 1) is a 10-year-old male with a history of exotropia, pulmonary valve stenosis, facial dysmorphism, learning disability, and ASD. He was the first child born to a non-consanguineous Caucasian couple from South Africa. Pregnancy was unremarkable. Delivery was induced at 36.5 weeks’ gestation due to oligohydramnios of unknown cause. Birth weight was 2353 g (10th percentile). An echocardiogram revealed mild pulmonary valve stenosis. Hearing and vision have been normal. At 7 years of age, weight, height, BMI, and head circumference were all at the 25th percentile. Dysmorphic features were present (Fig. 1). Development was notable for mild delay in independent walking (18 months) and more significant delay in expressive speech, with first clear words around 3½ years. He did not demonstrate craniosynostosis as might be suggested by the de novo frameshift mutation found in *SMAD6* (Supplemental information Table 1).

At age 6 years, he was diagnosed with a learning disability related to processing speed and working memory deficits, challenges with visual memory, and ASD. At 8 years old, a repeat assessment for educational planning showed superior verbal reasoning and average nonverbal reasoning skills and persistent deficits in processing speed and working memory. Psychological assessment results are summarized in Table 1.

**Table 1:** Summary of clinical features and psychological assessment data for the proband (22q11.2 triplication) and each of the proband’s family members (22q11.2 duplication)

	II-1 (proband)	I-1 (father)	I-2 (mother)	II-2 (sister)	II-3 (sister)
22q11.2 copy number	4	3	3	3	3
Age at last evaluation (years)	10	41	44	8	8
Sex	M	M	F	F	F
Physical/medical characteristics					
Pulmonary valve stenosis	+	–	–	–	–
Exotropia	+	–	–	–	–
Hooded eyelids	+	–	–	–	–
Epicanthal folds	+	–	–	–	–
Widely spaced eyes	+	–	–	–	–
Long, flat philtrum	+	+	–	–	–
Thin upper lip	+	–	–	–	–
Preauricular ear pits	+(bilateral)	+(unilateral)	–	+(bilateral)	+(bilateral)
Psychological diagnoses/assessments					
Learning disability	+	–	–	–	–
Autism spectrum disorder	+	–	–	–	–
Intelligence test (percentiles)	WISC-IV	WASI-II	WASI-II	WASI-II	WASI-II
Overall IQ	*Not calculated (**V > NV)	Average (66th)	Not calculated (**V > NV)	Average (73rd)	Average (68th)
* Subtest Scores*:					
Verbal reasoning (V)	Superior (91st)	Average (61st)	Average (39th)	Average (63rd)	Average (61st)
Non-verbal reasoning (NV)	Average (27th)	Average (66th)	Low Average (16th)	Average (61st)	Average (73rd)
Processing speed	Extremely low (2nd)	–	–	–	–
Working memory	Low average (9th)	–	–	–	–
Adaptive functioning: VABS-II (percentiles)	Daily living: average (50th) Communication (12th) & Socialization (9th): Below Average	n/a	n/a	Average in all domains	Average in all domains
Academic achievement: WJ-III (percentiles)					
Basic reading:	Borderline (3rd)	Average (57th)	Average (51st)	Average (62nd)	Average (71st)
Math reasoning:	Average (27th)	Average (47th)	Average (28th)	Average (50th)	Average (29th)
Attention deficit/hyperactivity symptomatology:	Elevated scores on inattention, hyperactivity & impulsivity	n/a	n/a	No concerns related to attention or hyperactivity	No concerns related to attention or hyperactivity
Connors-III (parent report)	Very elevated scores on learning problems & peer relations				
Autism spectrum disorder symptoms: SRS-2	Moderate deficits in reciprocal social behavior	n/a	n/a	Within normal limits	Within normal limits
Social communication questionnaire: SCQ (scores ≥ 15 indicate a possible ASD diagnosis)	Score = 11	n/a	n/a	Score = 0	Score = 0
Screen for depression: CDI-II (for children); BDI-II (for adults)	Average (no concerns; parent & self-report)	Mild symptoms endorsed	Mild symptoms endorsed	High average (88th) (self-report);Average (parent report)	Elevated (97th)(self-report); Average (parent report)
Screen for anxiety: MASC-II (for children); BAI (for adults)	Average (no concerns; parent & self-report)	Mild symptoms endorsed	Mild symptoms endorsed	Social anxiety score slightly elevated (self-report); Average (parent report)	Social anxiety score very elevated (self-report); Average (parent report)

Numbers in parentheses represent percentiles

*VABS-II* Vineland Adaptive Behavior Score, *SRS-2* social responsiveness questionnaire, *WASI-II* Wechsler Abbreviated Scale of Intelligence, *WISC-IV* Wechsler Intelligence Scale for Children, *WJ-III* Woodcock–Johnson Test of Achievement

*Due to significant discrepancy between verbal reasoning skills, nonverbal reasoning skills and processing speed and working memory skills, a full-scale IQ is not an accurate reflection of overall level of cognitive ability and was not computed

**Due to the significant discrepancy between the verbal and non-verbal reasoning skills a full-scale IQ is not an accurate reflection of overall level of cognitive functioning and was not computed

#### I-1: Father (duplication 22q11.2)

The father of the proband (I-1 in Fig. 1) is a 41-year-old man who denies medical problems. He attended regular classes at grade level in both elementary and secondary school. He is a vocational college graduate working full time. On examination, he had a left pre-auricular pit, a long, flat philtrum, and a wide uvula. Details of psychological assessment done at age 38 years are found in Table 1. On tests of intellectual functioning and academic skills, he scored within the average range.

#### I-2: Mother (Duplication 22q11.2)

The mother of the proband (I-2 in Fig. 1) is a 44-year-old woman who denies medical problems. As a child she was in regular classes at grade level. She is a vocational college graduate who formerly worked as a medical receptionist. On examination, no dysmorphic features were observed. Details of psychological assessment done at age 41 years are found in Table 1. On tests of intellectual functioning and academic skills, she scored within the average range for verbal reasoning and academic skills, and low average for perceptual reasoning skills. Clinically significant symptoms of anxiety and depression were endorsed on a self-report measure.

#### II-2 and II-3: Sisters (duplication 22q11.2)

The sisters of the proband (II-2 and II-3 in Fig. 1) are monozygotic twins. They have no significant past medical history and developmental milestones were met within expected age norms. They are currently in regular grade 3 functioning academically at grade level. At age 8 years, both have average growth parameters; both have bilateral preauricular ear pits but no other dysmorphic features. Details of psychological assessment at age 8 years are found in Table 1. Both girls have average overall intellectual abilities and academic skills, and both endorsed clinically elevated symptoms of social anxiety.

## Discussion

We present the first reported case of “double duplication” of the 22q11.2 region. Probands with this duplication have a wide range of phenotypes affecting physical and cognitive development, and the duplication is frequently inherited from a parent.^4^ Thus, it remains unclear whether increased dosage of this region truly causes a recognizable phenotype. Some authors have speculated that it could be a benign polymorphism or a risk variant which requires a “second hit” in order to result in a clinically significant phenotype.^8^ A second hit could, in theory, be an additional copy of the same region, assuming that one or more of the involved genes are dosage sensitive. The triplicated region in our proband contains 66 RefSeq genes, including *TBX1* and *CRKL*; cardiac defects in the 22q11.2 deletion syndrome have been attributed to haploinsufficiency of these two genes.^9, 10^ Our proband had pulmonic stenosis, while another triplication proband had sub-aortic stenosis and a ventricular septal defect.^11, 12^ Although experimental evidence is lacking, it is plausible that increased dosage of these genes could also predispose to aberrant cardiac development. The only other report of a proband with a 22q11.2 triplication, also (3:1), is an 8-year-old girl with a strikingly similar phenotype to our proband: learning difficulties, social immaturity, and similar dysmorphic features.^13^

Another possible reason for our proband’s phenotype, in particular his autistic features, is the contribution of additional mutations in autism candidate genes. We investigated this by analyzing the genome sequence of the proband and his parents using our variant pipeline optimized for identification of ASD-related genes^14^; however, we are yet to identify any convincing additional mutations that would be clinically characterized as being pathogenic for ASD. Alternatively, it is possible that increased copy number at 22q11.2 could contribute to vulnerability to ASD; at least one study has reported a relatively high prevalence of autism in probands with 22q11.2 duplication.^15^ From a genomic point of view, this case could be considered as an example of a recessive disorder caused by copy number gains, since the parents carrying three copies are normal, but the proband carrying four copies presents with a phenotype.

In conclusion, we describe in detail the phenotype of a boy with four copies of the 22q11.2 region, and his four first-degree relatives who carry the duplication (three copies) to illustrate the effects of increased copy number of the 22q11.2 region. This family provides support for the view that the 22q11.2 duplication may not be pathogenic on its own and require additional rare and/or common variants, perhaps in unique combinations, to express phenotype.^16–20^ However, a triplication (either by double duplication, as in our proband, or by expansion of a parental duplication) of the region may result in a phenotype affecting cardiovascular development, facial appearance, cognition, and social communication.

## Methods

CMA by array CGH (4 × 180 K ISCA design, Oxford GeneTechnology, Oxford, UK) was performed on all five family members. The proband and both parents also had FISH testing with TUPLE/HIRA1 probe (Vysis, Abbott Park, Illinois, USA). As part of a separate research study, the proband and both parents had WGS as described recently with the data available in the Autism Speaks-supported MSSNG whole genome sequence and phenotype database.^14, 21^ All sequence data can be accessed through the MSSNG database sponsored by Autism Speaks on Google Genomics (https://www.mss.ng). The proband and family members underwent psychological assessment and examination by a clinical geneticist. The methods were performed in accordance with relevant guidelines and regulations and approved by the Research Ethics Board at the Hospital for Sick Children (Toronto, Canada). The parents provided written informed consent for participation and publication of photographs.

### Data availability

The data, as part of a larger autism whole-genome sequencing project, are available in the MSSNG database on Google Genomics (for access see http://www.mss.ng/researchers).

## Electronic supplementary material

Supplementary Figure 1

Supplementary Figure 2

Supplementary Table 1
