# Supplementary figures and images for: Atypical autism in a boy with double duplication of 22q11.2: implications of increasing dosage
Source: NPJ Genom Med. 2017 Sep 28;2:28. doi: 10.1038/s41525-017-0031-6 (PMC5677976; doi:10.1038/s41525-017-0031-6)

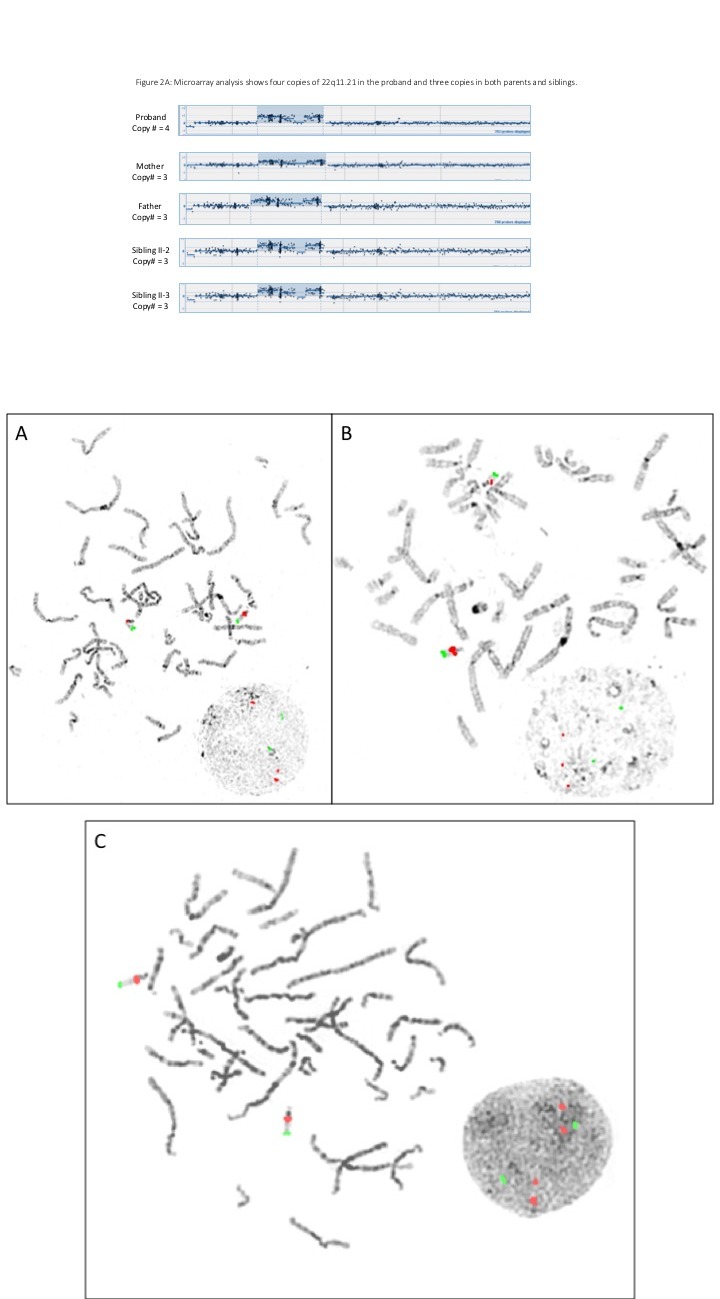

Supplement: Supplementary file 1 — Supplementary Figure 1 [file 41525_2017_31_MOESM1_ESM.jpg]
