# Supplementary material for: Atypical autism in a boy with double duplication of 22q11.2: implications of increasing dosage
Source: NPJ Genom Med. 2017 Sep 28;2:28. doi: 10.1038/s41525-017-0031-6 (PMC5677976; doi:10.1038/s41525-017-0031-6)
Supplement: Supplementary file 2 — Supplementary Figure 2 [file 41525_2017_31_MOESM2_ESM.docx]

Supplementary Figure 2: Distribution of allele ratios of informative SNPs within the 22q11.2 duplication

The distribution of allele ratio of SNPs within the 22q11.2 duplicated region that were informative (both parents were homozygous with different genotypes and the proband was heterozygous) is shown. The unimodal distribution clustered around 0.5 indicates equal contribution of parental alleles.
